# Association between RUNX3 promoter methylation and gastric cancer: a meta-analysis

**DOI:** 10.1186/1471-230X-11-92

**Published:** 2011-08-25

**Authors:** Xiao-yuan Fan, Xin-lei Hu, Tie-mei Han, Na-na Wang, Yi-miao Zhu, Wen Hu, Zhen-hua Ma, Chen-jing Zhang, Xiang Xu, Zai-yuan Ye, Chun-mao Han, Wen-sheng Pan

**Affiliations:** 1 Department of Gastroenterology, Second Affiliated Hospital, Zhejiang University, School of Medicine, 88 Jiefang Road, Hangzhou, China; 2 Department of Orthopaedics, Second Affiliated Hospital (Binjiang Branch), Zhejiang University, School of Medicine, 88 Jiefang Road, Hangzhou, China; 3 Department of Pharmacy, Second Affiliated Hospital, Zhejiang University, School of Medicine, 88 Jiefang Road, Hangzhou, China; 4 Department of General Surgery, Zhejiang Provincial People's Hospital, 158 Shangtang Road, Hangzhou, China; 5 Department of Burns, Second Affiliated Hospital, Zhejiang University, School of Medicine, 88 Jiefang Road, Hangzhou, China

## Abstract

**Background:**

Runt-related transcription factor 3 (RUNX3) is a member of the runt-domain family of transcription factors and has been reported to be a candidate tumor suppressor in gastric cancer. However, the association between RUNX3 promoter methylation and gastric cancer remains unclear.

**Methods:**

We systematically reviewed studies of RUNX3 promoter methylation and gastric cancer published in English or Chinese from January 2000 to January 2011, and quantified the association between RUNX3 promoter methylation and gastric cancer using meta-analysis methods.

**Results:**

A total of 1740 samples in 974 participants from seventeen studies were included in the meta-analysis. A significant association was observed between RUNX3 promoter methylation and gastric cancer, with an aggregated odds ratio (OR) of 5.63 (95%CI 3.15, 10.07). There was obvious heterogeneity among studies. Subgroup analyses (including by tissue origin, country and age), meta-regression were performed to determine the source of the heterogeneity. Meta-regression showed that the trend in ORs was inversely correlated with age. No publication bias was detected. The ORs for RUNX3 methylation in well-differentiated *vs *undifferentiated gastric cancers, and in intestinal-type *vs *diffuse-type carcinomas were 0.59 (95%CI: 0.30, 1.16) and 2.62 (95%CI: 1.33, 5.14), respectively. There were no significant differences in RUNX3 methylation in cancer tissues in relation to age, gender, TNM stage, invasion of tumors into blood vessel or lymphatic ducts, or tumor stage.

**Conclusions:**

This meta-analysis identified a strong association between methylation of the RUNX3 promoter and gastric cancer, confirming the role of RUNX3 as a tumor suppressor gene.

## Background

Gastric cancer is the second most common gastrointestinal tumor worldwide. Although its incidence continues to decline year by year, it remains the second most common causes of cancer-related deaths [1,2].

Runt-related transcription factor 3 (RUNX3) is a member of the runt domain family of transcription factors, also known as polyomavirus enhancer-binding protein 2 (PEBP2)/core binding factors (CBF) [3-5]. RUNX3 is a known regulator of major developmental pathways, and has recently been reported as a candidate tumor suppressor [3-5]. RUNX3 is located on human chromosome region 1p36 and plays an important role in the transforming growth factor (TGF)-β signaling pathway. This may occur partly through interaction with FoxO3a/FKHRL1, both of which are indispensable for the activation of the pro-apoptosis protein Bim (Bcl-2-interacting mediator of cell death), or by cooperating with SMAD protein family members to induce the TGF-β/SMAD pathway in other ways [6,7]. The RUNX3 gene is regulated by three main mechanisms: loss of heterozygosity (LOH), protein mislocalization, and promoter methylation [3,5,8,9]. Silencing of the promoter region CpGs of regulated genes via hypermethylation is recognized as one of the mechanisms responsible for loss-of-gene-function in Knudson's two-hit tumor progression hypothesis[10]. Numerous studies using cell lines, knockout animals, and primary human cancer samples have demonstrated a crucial role for RUNX3 not only in normal development, but also in neoplasias, especially stomach cancers [3,5,11]. Previous studies have reported the involvement of RUNX3 promoter methylation in many cancers, including colorectal [12,13], gastric [14], bladder [15], breast [16], lung [17], oral [18], and nasopharyngeal cancers [19]. However, the role of RUNX3 in the regulation of gastric epithelial cell growth and its tumor suppressor activity in gastric cancer remain to be clarified. Levanon and colleagues questioned the causal relationship between loss of RUNX3 expression and gastric cancer, and reported that RUNX3-deficient mice did not develop gastric hyperplasia or gastric tumors [20,21]. Carvalho et al. refuted a tumor suppressor role for the RUNX3 gene in early-onset gastric carcinomas, on the basis of its lack of expression in histological non-neoplastic gastric epithelium, although at least two copies of the gene were present in the vast majority of cells analyzed [22]. However, associations between RUNX3 promoter methylation and gastric cancer have mostly been investigated in studies with small sample sizes, low statistical power, specific ethnic backgrounds, or other limitations in study design, leading to conflicting results.

Meta-analysis is a well-established method for quantifying gene-disease associations more precisely, by using all the available published data to increase the statistical power [23]. We therefore conducted a meta-analysis using all available related studies to better define the association between RUNX3 promoter methylation and gastric cancer.

## Methods

### Search strategy

This pooled study involved searching a range of computerized databases, including Medline, Blackwell, Cochrane Central, Web of Science, and Ovid, for articles published in English or Chinese from January 2000 to January 2011. The study used a subject and text word strategy with (RUNX3 OR AML2 OR CBFA3 OR PEBP) AND ((gastric OR stomach) AND (cancer OR neoplasm)) as the primary search terms. The search strategy was tailored to each database to ensure that the search was comprehensive and not limited to randomized controlled trials.

### Selection of studies and data extraction

Three independent reviewers (Fan, Hu and Pan) screened the titles and abstracts identified by the electronic search to identify relevant studies. Relevant articles were further examined to see if they met the inclusion criteria. The reference sections of all retrieved articles were searched to identify further relevant articles. A citation search was carried out on all included articles using the Science Citation Index. Potentially relevant papers were obtained and the full text articles were screened for inclusion by two independent reviewers (Fan, Hu). Disagreements were resolved by discussion. Included studies were summarized in data extraction forms. Authors were contacted when relevant data were missing.

The name of the first author, year of publication, origin of the study patients, sample size, and methylation status of the RUNX3 promoter in human gastric cancer and normal or control tissues were extracted. The inclusion criteria were as follows: the patients had to be diagnosed with gastric cancer and the studies had to have RUNX3 gene promoter methylation data from tissue samples. The following types of studies were excluded: animal experiments, case reports, review articles or meta-analyses, and studies with insufficient data.

### Data analysis and synthesis

Data were analyzed mainly using STATA Software (Stata/SE 11.0 for Windows, StataCorp LP). The strength of association was expressed as pooled odds ratio (OR) with corresponding 95% confidence intervals (CI). Data were extracted from the original studies and recalculated if necessary. The data were pooled using the DerSimonian and Laird random effects model [24], which takes account of both within-study and between-study variations. A two-sided *P *≤ 0.05 was considered significant. Heterogeneity was tested using the I^2 ^statistic with values >50% and χ^2 ^test with *P *≤ 0.05 indicating strong heterogeneity between the studies. τ^2 ^was used to determine how much heterogeneity was explained by subgroup differences [25]. If the heterogeneity was significant, Galbraith plot and meta-regression analyses were employed to analyze the sources of the heterogeneity. Subgroup analyses of the ORs of RUNX3 promoter methylation in cancer tissue versus normal tissue were performed according to control types (autogenous and heterogeneous), patient origins (Europe-America, China, Japan, and Korea) and age categories (<60, between 60 and 65, and ≥65). Sensitivity analyses were performed to assess the contributions of single studies to the final results. Possible bias was analyzed by funnel plot. If bias was suspected, the meta-trim method was used to re-estimate the effect size. Differences in methylation status of cancer tissues were also analyzed in relation to age (≤60, >60), gender (male, female), metastases, and histopathological cancer type.

## Results

### Study characteristics

The electronic search strategy identified 132 potentially relevant articles, which were further screened for inclusion on the basis of their titles, abstracts, full texts, or a combination of these. The electronic search was supplemented from reference lists of relevant articles including reviews, and by discussion with experts.

A total of 55 full-text articles were retrieved, of which 38 were excluded. The remaining 17 studies included data on the relationship between RUNX3 gene promoter methylation and gastric cancer, and these studies were pooled for analysis (Table 1) [14,26-41]. Sixteen of the included articles were written in English, and one was written in Chinese. The main characteristics of each study are shown in the supplementary data (Table 1).

**Table 1:** Basic characteristics of the included studies

Study/Country	Mean/median age (range) (years)	Gender (M/F)	Patients (M+/M-)	Control (M+/M-)	Methods	Primary Aim	Methylation site	RUNX3 Expression
Waki et al (2003)[26]/Japan	64.3 (0.7-89)	68/25	42/51	7/86A 4/15H	MSP, RT-PCR	Clarify the physiological consequence of DAP-kinase and RUNX3 age-related methylation in gastric epithelia	CpG islands	+
Oshimo et al (2004)[14]/Japan	68.8 (38-87)	56/24	57/23	38/7H	MSP, qRT-PCR	Loss of RUNX3 expression by promoter hypermethylation in Gastric Carcinoma	CpG islands	+
Nakase et al (2005)[27]/Japan	65.4	81/32	14/8	6/16A	MSP, qRT-PCR	Determine whether alteration of RUNX3 gene expression could be detected in the normal-looking gastric remnant mucosa stomach after distal gastrectomy for peptic ulcer or gastric cancer	promoter hypermethylation	+
Homma et al (2006)[28]/USA	63 (30-85)	28/17	43/2	43/2A	MSP	Clarify how methylation spreads within the CpG island	region NO.1 at CpG islands	-
So et al (2006)[29]/Japan	64 (30-82)	17/9	11/5	6/20A	MSP, microarray	Use microarray-based methylation assay to assess gene methylation	CpG islands	-
Mitsuno et al (2007)[30]/Japan	63.2 (26-86)	29/9	18/20	Not reported	MSP	Determine whether DNA methylation in six cancer-related genes affects recurrence of gastric cancer in patients who received 5-fluorouracil-based adjuvant chemotherapy	CpG islands	-
Gargano et al (2007)[31]/Italy	Not reported	65/35	22/18	2/38A	MSP	Investigate a possible relationship between the RUNX3 promoter methylation, nuclear microsatellite instability and mitochondrial microsatellite instability	CpG islands	-
Fujii et al (2008)[32]/Japan	62.8 (52-77)	8/9	11/6	Not reported	MSP, ChIP, RT-PCR	Enhancer of Zeste Homologue 2 (EZH2) down-regulates RUNX3 by increasing histone H3 methylation	CpG islands	+
Kitajima et al (2008)[33]/Japan	65.71	34/23	30/27	10/47A	MSP, PCR	Determine the Helicobacter pylori infection as an independent risk factor for Runx3 methylation in gastric cancer	CpG islands	+
Li et al (2008)[34]/China	62 (35-78)	23/17	22/18	0/40A	MSP	Analyze the relationships among the aberrant methylation of RUNX3 gene promoter, the RUNX3 protein expression and clinicopathological features in gastric cancer	CpG islands	+
Song et al (2008)[35]/Korea	64	50/29	26/53	9/70A	MSP	Determine the methylation of RUNX3 promoter and the association between RUNX3 methylation and the clinicopathological characteristics of patients with gastric carcinoma	CpG islands	-
KIM et al (2009)[36]/Republic of Korea	57.7	53/21	18/56	2/61A	MSP	Comparison of DNA methylation between primary and metastatic gastric carcinoma	promoter	-
Zou et al (2009)[37]/China	65.15	40/17	14/2	0/20H	MSP	Determine the methylation frequency of 5 genes, including p16, Runx3, MGMT, DAPK, and RASSF1A	CpG islands	+
Chen et al (2010)[38]/China	53 (20-78)	20/50	28/42	2/68A	MSP, RT-PCR	Hypermethylation downregulates Runx3 gene expression and its restoration suppresses gastric epithelial cell growth by inducing p27 and caspase3 in human gastric cancer	CpG islands	+
Hiraki et al (2010)[39]/Japan	68.6 (45-88)	30/19	28/21	14/35A	q-MS, qRT-PCR	Determine whether gene methylation is a novel diagnostic marker for micrometastasis to the lymph nodes (LNs) in gastric cancer	Promoter hypermethylation	+
Hu et al (2010)[40]/China	64.1	97/26	68/55	12/111A	MSP, RT-PCR	Pathobiologic implications of methylation and expression status of Runx3 and CHFR genes in gastric cancer	CpG islands	+
Mikata et al (2010) [41]/Japan	70 (56-85)	14/7	10/11	4/17A	MS, qRT-PCR, RT-PCR	BCL2L10 hypermethylation in gastric cancer and its correlation with RUNX3	CpG islands	-

A: Autologous control, the control tissues from the patients themselves; H: Heterogeneous control, the control tissues from other individuals. M+: The number of tissues with methylation; M-: The number of tissues with unmethylation.

**Figure 1:**
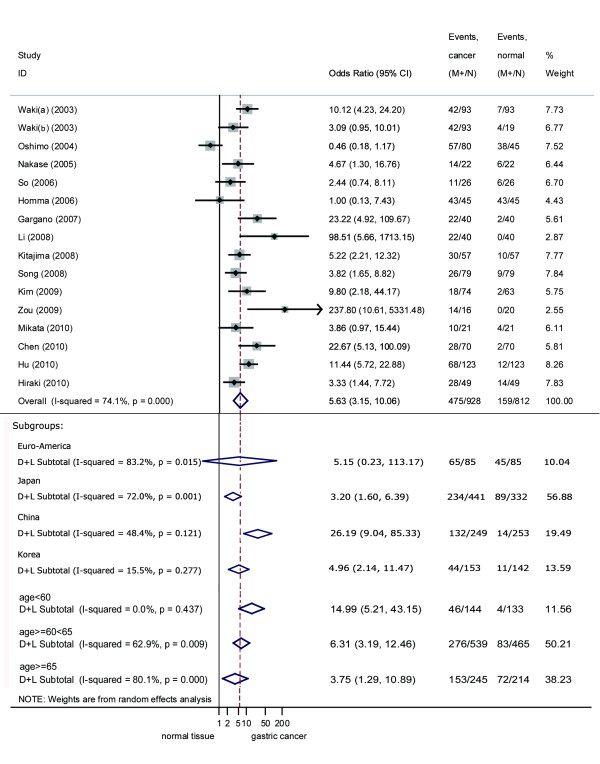
**Forest plot of RUNX3 promoter methylation in cancer tissue vs normal tissue and subgroup analyses**.

Among the 17 retrieved articles, 16 observations in 15 studies used methylation-specific polymerase chain reaction to explore RUNX3 promoter methylation in gastric cancer tissues (n = 928) and normal tissues (n = 812), with a total sample size of 1740 cases. The total number of participants is 974 from 17 articles including 890 patients from 17 articles and 84 controls from 3 articles which provided heterogeneous control groups. The pooled OR for RUNX3 methylation in cancer tissues compared with normal tissues was 5.63 (95%CI 3.15-10.06, z = 5.82, *P *< 0.0001), indicating an increased likelihood of methylation in gastric cancer tissue, compared with normal tissue (Figure 1).

### Subgroup analysis and meta-regression

The OR in the autologous tissue subgroup was 6.42 (95%CI: 4.07, 10.11; *P *< 0.0001) and that in the heterogeneous tissue subgroup was 4.44 (95%CI: 0.35, 56.70; *P *= 0.251) (Table 2). Tissue subgroup analysis showed that the incidence of methylation in autologous cancer tissues was higher than that in normal tissues, but there was no obvious difference between autologous and heterogeneous tissues. The difference was not deemed significant by the random effects method, because of the existing heterogeneity (I^2 ^= 89.30%, χ^2 ^= 18.69; *P *< 0.0001) (Table 2). The OR for the Japanese subgroup was 3.20 (95%CI: 1.60, 6.39; *P *= 0.001), for the European-American subgroup was 5.15 (95%CI: 0.23, 113.17; *P *= 0.251), for the Chinese subgroup was 26.19 (95%CI: 8.04, 85.33; *P *< 0.0001), and for the Korean subgroup was 4.96 (95%CI: 2.14, 11.47; *P *< 0.0001) (Table 2, Figure 1). Heterogeneity also existed within patients of each origin, especially in the European-American subgroup (I^2 ^= 83.20%, χ^2 ^= 5.95; *P *= 0.015), which changed the OR from significant (fixed) to non-significant (random) (Table 2, Figure 1). The OR for the age <60 group was 15.00 (95%CI: 5.21, 43.15; *P *< 0.0001), that for the 60-65 subgroup was 6.31 (95%CI: 3.19, 12.46; *P *< 0.0001), and that for the ≥65 subgroup was 3.75 (95%CI: 1.29, 10.89; *P *= 0.015) (Table 2, Figure 1).

**Table 2:** Subgroup analysis


**Group**	**Cancer**		**Normal **		**Sum**	**M-H pooled OR**			**D+L pooled OR**			**Heterogeneity**			**τ^2^**
						
	**M+**	**N**	**M+**	**N**		**OR (95%CI)**	**z**	**p**	**OR(95%CI)**	**z**	**P**	**χ^2^**	**I^2 ^(%)**	**P**	
						
Total	475	928	159	812	1740	5.52(4.30, 7.10)	13.3	< 0.0001	5.63(3.15,10.07)	5.82	< 0.0001	57.91	74.1	< 0.0001	0.94

Tissue subgroup															
Autologous tissue	362	739	117	728	1467	7.15(5.36, 9.53)	13.4	< 0.0001	6.42(4.07,10.11)	8	< 0.0001	24.12	50.02	0.02	0.32
Heterogeneous tissue	113	189	42	84	273	5.52(4.30, 7.10)	2.22	0.026	4.44(0.35,56.70)	1.15	0.251	18.69	89.3	< 0.0001	4.21

Origin subgroup															
Japan	234	441	89	332	773	3.20(2.30, 4.47)	6.86	< 0.0001	3.20(1.60,6.39)	3.29	0.001	25.03	72	0.001	0.7
Euro-America	65	85	45	85	170	8.12(2.76, 23.88)	3.8	< 0.0001	5.15(0.23,113.17)	1.04	0.299	5.95	83.2	0.015	4.14
China	132	249	14	253	502	18.44(10.30, 33.03)	9.8	< 0.0001	26.19(8.04,85.33)	5.42	< 0.0001	5.82	48.4	0.121	0.66
Korea	44	153	11	142	295	5.10(2.48, 10.47)	4.42	< 0.0001	4.96(2.14,11.47)	3.74	< 0.0001	1.18	15.5	0.277	0.07

Age subgroup															
age < 60	46	114	4	133	247	15.25(5.33, 43.61)	5.08	< 0.0001	15.00(5.21,43.15)	5.02	< 0.0001	0.6	< 0.001	0.437	< 0.001
60≤age < 65	276	539	83	465	1004	7.41(5.16, 10.62)	10.9	< 0.0001	6.31(3.19,12.46)	5.3	< 0.0001	18.88	62.9	0.009	0.54
age≥65	153	245	72	214	459	2.93(1.98, 4.32)	5.39	< 0.0001	3.75(1.29,10.89)	2.43	0.015	25.18	80.1	< 0.0001	1.31

M+: The number of tissues with methylation; N: Number of the total observations.

Heterogeneity existed in all studies (I^2 ^= 74.10%, χ^2 ^= 57.91, *P *< 0.0001) (Table 2). We therefore performed further analyses using the meta-regression method with the Knapp-Hartung modification. The restricted maximum likelihood method was used to estimate between-study variance.

The results of meta-regression indicated that the trend in ORs was inversely correlated with age, which accounted for some of the heterogeneity (coefficient = -0.16, *P *= 0.042, adjusted R^2 ^= 44.47%, Table 3). However, other factors such as sample size, year of publication, proportion of males, and the origin of the patients could not explain the heterogeneity (Table 3).

**Table 3:** Meta-regression

Sources	Coefficient (95%CI)	t	P	τ^2^	I^2 ^Res (%)	Adjusted R^2 ^(%)
Sample size	-0.01 (-0.03, 0.01)	-1.04	0.314	1.07	75.33	-7.96
Year of publication	0.19 (-0.07, 0.46)	1.54	0.145	0.90	71.95	9.9
Proportion of males	-2.91 (-9.67, 3.84)	-0.93	0.370	1.03	75.5	-3.89
Control type	-1.06 (-2.87, 0.76)	-1.25	0.233	0.70	65.97	29.99
Age	-0.16 (-0.31, -0.01)	-2.22	0.042	0.52	63.91	44.47
Origin of the patients	-0.57 (-1.16, 0.03)	-2.05	0.060	0.66	66.74	33.92

### Bias analysis and robust estimation of pooled OR

**Figure 2:**
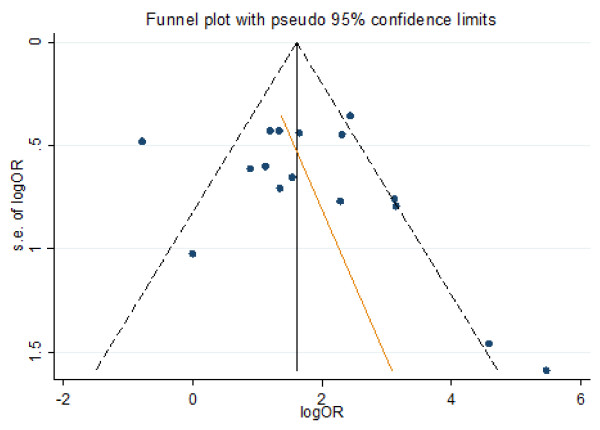
**Funnel plot**. The results of the funnel plot showed that the standard errors in four of the studies exceeded the 95% confidence limit, and the Egger's regression line showed that the smaller studies tend to report more positive association of RUNX3 promoter methylation with gastric cancer but was not significant (P = 0.593).

A funnel plot of methylation status of gastric cancer tissue versus normal tissue showed that four studies exceeded the 95% confidence limits (Figure 2). We also performed sensitivity analyses to determine the effects of omitting a single study on the overall effect; omission of a single study changed the overall OR from 5.15 (2.85, 9.29) to 6.09 (3.37, 11.0) using the random method, suggesting that there was no single sensitive study (Table 4). Trim and fill analysis was performed using the random effects model. The pooled OR was 4.67 (95%CI: 2.57, 8.49; *P *< 0.0001) indicating a positive association between RUNX3 methylation and gastric cancer.

**Table 4:** Sensitivity analysis

Study omitted	Estimate OR	95% CI	
		
		Lower	Upper
Waki(a) (2003)	5.40	2.90	10.06
Waki(b) (2003)	5.92	3.19	10.98
Oshimo (2004)	6.55	4.12	10.43
Nakase (2005)	5.74	3.09	10.66
Homma (2006)	6.09	3.37	11.00
So (2006)	6.00	3.25	11.08
Gargano (2007)	5.16	2.85	9.32
Kitajima (2008)	5.74	3.03	10.86
Li (2008)	5.15	2.90	9.14
Song (2008)	5.89	3.12	11.13
Kim (2009)	5.46	2.97	10.04
Zou (2009)	5.08	2.89	8.93
Hu (2010)	5.30	2.86	9.83
Chen (2010)	5.15	2.85	9.29
Hiraki (2010)	5.95	3.16	11.20
Mikata (2010)	5.80	3.14	10.73
Combined	5.63	3.15	10.06

### Comparators in cancer tissue

There were no significant differences in RUNX3 methylation status in cancer tissue in relation to age, gender, TNM stage, invasion of tumors into blood vessels or lymphatic ducts, or tumor stage, or tumor metastases (Data not shown).

The overall results demonstrated that RUNX3 methylation was more frequent in intestinal-type, compared with diffuse-type gastric carcinomas (OR = 2.62, 95%CI: 1.33, 5.14). Undifferentiated gastric cancer also had a higher methylation OR than well-differentiated cancer tissue (OR = 0.59, 95%CI: 0.30, 1.16) (Data not shown).

## Discussion

The role of the RUNX3 gene in gastric cancer is controversial [3,20,22,42,43]. Inactivation of the RUNX3 gene can be caused by LOH, promoter hypermethylation, or protein mislocalization. Methylation of the RUNX3 promoter is one of the most common aberrant methylation events in cancer [44]. We therefore performed a meta-analysis to quantify the association between RUNX3 promoter methylation and gastric cancer.

The overall OR for methylation status in gastric cancer versus normal gastric tissue was 5.63 (3.15, 10.07), using a random effects model on pooled data from 16 observations in 15 studies. Subgroup analysis showed an OR in the heterogeneous tissue-origin subgroup of 4.44 (0.35, 56.70), which was significant in the fixed, but not in the random effects model. This discrepancy may be a result of the smaller number of studies analyzed. The ORs also differed in subgroups with different ethnic origins: the OR in the Chinese subgroup was 26.19 (95%CI: 8.04, 85.33), followed by the European-American subgroup (OR = 5.15, 95%CI: 0.23, 113.17), the Korean subgroup (OR = 4.96, 95%CI: 2.14, 11.47), and the Japanese subgroup (OR = 3.20, 95%CI: 1.60, 6.39). The ORs for the different age subgroups were 15.00 (95%CI: 5.21, 43.15) for age <60, 6.31 (95%CI: 3.19, 12.46) for age 60-65, and 3.75 (95%CI: 1.29, 10.89) for age ≥ 65. Heterogeneity within the studies was demonstrated by χ^2^and I^2 ^tests and meta-regression was therefore used to determine the sources of the heterogeneity. This showed that the trend in ORs was inversely correlated with age, which accounted for at least some of the heterogeneity (coefficient = -0.16, P = 0.042, adjusted R^2 ^= 44.47%). These results were consistent with the results of subgroup analysis according to age. The incidence of age-related methylation in most organs was in accordance with the reported incidences of methylation in their malignant counterparts [45]. RUNX3 methylation was reported to occur preferentially in the lower third portion of the stomach in individuals older than 70 years [45]. We found that the ORs for RUNX3 methylation decreased from 15.00 in the younger age group, through 6.31, to 3.75 in the oldest age group. The coefficient for age was calculated to be -0.16 by meta-regression analysis, indicating that the tendency for RUNX3 methylation decreased with advancing age. To the best of our knowledge, the current study is the first to report this finding. However, there was no significant difference in RUNX3 promoter methylation status of cancer tissues between older (more than 60) and younger (less than 60) individuals. This suggests that RUNX3 methylation was not correlated with age in gastric cancer tissue. However, in accordance with the previous study, RUNX3 methylation did decrease with advancing age in normal gastric mucosa [45]. The negative correlation between RUNX3 methylation and age suggests that the influence of RUNX3 methylation on gastric cancer is reduced in older individuals.

Other factors including sample size, year of publication, proportion of males, and the origins of the patients were not identified as sources of heterogeneity by meta-regression analysis.

Meta-analysis of small studies may result in biased results. The funnel plot showed that the standard errors in four of the studies exceeded the 95% confidence limits; furthermore, the asymmetry test of the funnel plot by Egger's regression method showed that the smaller studies reported more positive results, raising the suspicion of bias among the studies. To produce a more robust estimation, we performed sensitivity analysis using the random effects model and were unable to identify any individual sensitive study with a strong influence on the pooled results. Trim and fill tests were performed using the random effects model, and two virtual studies were filled. The overall OR of the trim and fill method was 4.67, which was slightly smaller than that of the crude meta-analysis, but it was still significant, indicating a strong association between RUNX3 promoter methylation and gastric cancer.

There were no significant differences in RUNX3 methylation in cancer tissues in relation to gender, TNM stage, invasion of tumors into vessels or lymphatic ducts, or tumor stage. Although some studies have reported significant differences in methylation status and protein expression of RUNX3 in relation to tumor invasion depth [46], the overall results of the current study failed to support the existence of such a relationship. Previous studies have also reported increased methylation of RUNX3 in stage I and II gastric cancers, suggesting that the RUNX3 gene contributes to gastric cancer development [38]. However, the results of the current meta-analysis did not support this assumption. Other factors, such as tumor invasion of blood vessels, lymph nodes or lymphatic ducts, and tumor metastases, also demonstrated no relation with RUNX3 methylation.

The aggregated results found that RUNX3 methylation was more frequent in intestinal-type compared with diffuse-type gastric carcinomas, suggesting that inactivation of RUNX3 might play a more significant role in the development of intestinal-type gastric carcinomas.

Analysis of the pooled data also showed that undifferentiated gastric cancers had a higher methylation OR than well-differentiated cancer tissues. This suggests that RUNX3 promoter methylation or down-regulation of the RUNX3 gene may be related to poor prognosis, as suggested in previous studies [47,48].

## Conclusions

In conclusion, this meta-analysis of pooled data provides additional evidence to support a strong association between methylation of the RUNX3 promoter and gastric cancer. This association depended on patient origin and the controls used, and further studies are needed to explore these aspects. Younger individuals had higher RUNX3 methylation rates than older individuals. RUNX3 methylation was also associated with histological type and differentiation state of the gastric cancer. However, gender, TNM stage, invasion of tumors into blood vessels or lymphatic ducts, and tumor stage showed no significant associations with RUNX3 methylation in gastric cancer tissues.

## Abbreviations

RUNX3: Runt-related transcription factor 3; PEBP2: polyomavirus enhancer binding protein 2; CBF: core binding factors; TGF-β: transforming growth factor-β; LOH: loss of heterozygosity; OR: odds ratio; CI: confidence interval; TNM: tumor: node: metastasis.

## Competing interests

The authors declare that they have no competing interests.

## Authors' contributions

All authors have made substantial contributions to this article: WP, XX, ZY and CH contributed to the conception, design and final approval of the submitted version. XF and XH contributed to the analysis and interpretation of data, and drafting of the article. TH, NW and YZ contributed to the acquisition of data and revision of the article. WH, ZM and CZ contributed to the acquisition of data and discussion of the article design. All authors read and approved the final manuscript.

## Pre-publication history

The pre-publication history for this paper can be accessed here:

http://www.biomedcentral.com/1471-230X/11/92/prepub
